# ROCK Inhibition May Stop Diabetic Kidney Disease

**DOI:** 10.31662/jmaj.2020-0014

**Published:** 2020-06-19

**Authors:** Keiichiro Matoba, Yusuke Takeda, Yosuke Nagai, Yasushi Kanazawa, Daiji Kawanami, Tamotsu Yokota, Kazunori Utsunomiya, Rimei Nishimura

**Affiliations:** 1 Division of Diabetes, Metabolism, and Endocrinology, Department of Internal Medicine, The Jikei University School of Medicine, Tokyo, Japan; 2 Department of Endocrinology and Diabetes Mellitus, Fukuoka University School of Medicine, Fukuoka, Japan; 3 Center for Preventive Medicine, The Jikei University School of Medicine, Tokyo, Japan

**Keywords:** ROCK, Rho-kinase, diabetes, diabetic kidney disease

## Abstract

Diabetic kidney disease (DKD) is the leading cause of end-stage renal disease and is strongly associated with cardiovascular mortality. Given the pandemic of obesity and diabetes, the elucidation of the molecular underpinnings of DKD and establishment of effective therapy are urgently required. Studies over the past decade have identified the activated renin-angiotensin system (RAS) and hemodynamic changes as important therapeutic targets. However, given the residual risk observed in patients treated with RAS inhibitors and/or sodium glucose co-transporter 2 inhibitors, the involvement of other molecular machinery is likely, and the elucidation of such pathways represents fertile ground for the development of novel strategies. Rho-kinase (ROCK) is a serine/threonine kinase that is under the control of small GTPase protein Rho. Many fundamental cellular processes, including migration, proliferation, and survival are orchestrated by ROCK through a mechanism involving cytoskeletal reorganization. From a pathological standpoint, several analyses provide compelling evidence supporting the hypothesis that ROCK is an important regulator of DKD that is highly pertinent to cardiovascular disease. In cell-based studies, ROCK is activated in response to a diverse array of external stimuli associated with diabetes, and renal ROCK activity is elevated in the context of type 1 and 2 diabetes. Experimental studies have demonstrated the efficacy of pharmacological or genetic inhibition of ROCK in the prevention of diabetes-related histological and functional abnormalities in the kidney. Through a bird’s eye view of ROCK in renal biology, the present review provides a conceptual framework that may be widely applicable to the pathological processes of multiple organs and illustrate novel therapeutic promise in diabetology.

## 1. Introduction

Diabetic kidney disease (DKD) remains the leading cause of end-stage renal disease and is associated with increased morbidity and mortality. The reasons for the pandemic of DKD include the aging of the population and the increase in patients with diabetes as well as obesity. DKD is characterized by functional and structural changes in the glomeruli, including glomerular hyperfiltration, mesangial expansion, thickening of the glomerular basement membrane, and podocyte loss. The denouement of these abnormalities is glomerular sclerosis accompanied by albuminuria and a decline in the glomerular filtration rate (GFR). Emerging evidence suggests a strong link between albuminuria and the cardiovascular risk in patients with DKD; an increase in albuminuria is not only a feature of DKD but also an independent risk factor for cardiovascular events (*i.e.*, reno-cardiac syndrome) ^(1)^. Also, annual cardiovascular mortality rates were found to increase with progression to renal failure in patients with type 2 diabetes ^(2)^. Therefore, it is important to design specific therapies for DKD, which requires defining the molecular mechanisms that drive DKD.

Pervasive programs are known to unleash during the progression of DKD, including the activation of the renin-angiotensin system (RAS) and hemodynamic changes ^(3)^. While there is no doubt that interventions for these abnormalities with angiotensin-converting enzyme inhibitors/angiotensin receptor blockers and sodium glucose co-transporter 2 inhibitor are critical for preventing the progression of DKD ^(4), (5)^, the current standards of care do not eliminate the risk of DKD. Given the limited agents for inhibiting the progression of DKD, there has been an ongoing effort to unravel the biological mechanisms responsible for the renal damage and to develop effective therapeutic approaches.

Experimental work over the past decade has demonstrated key roles of Rho-kinase (ROCK) in the initiation and progression of diabetic vascular complications. This review will focus on the pathogenic roles of ROCK in DKD and other vascular diseases.

## 2. ROCK: General Considerations

ROCK is a serine/threonine kinase that acts as an effector of the small GTPase Rho (meaning “Ras homolog”). Binding of ROCK to the active GTP-bound form of Rho endows eukaryotes with the ability to regulate several essential cellular functions such as the modulation of cell shape, motility, proliferation, and the expression of various genes. A large body of work has focused on the role of ROCK in cytoskeletal arrangement and demonstrated that the major downstream effectors of ROCK include myosin phosphatase target subunit 1, myosin light chain (MLC), and LIM kinase, thereby regulating actin dynamics. The formation of stress fibers and focal adhesion complexes mediated by MLC activation is one of the first functions of ROCK to be identified.

**Figure 1.**
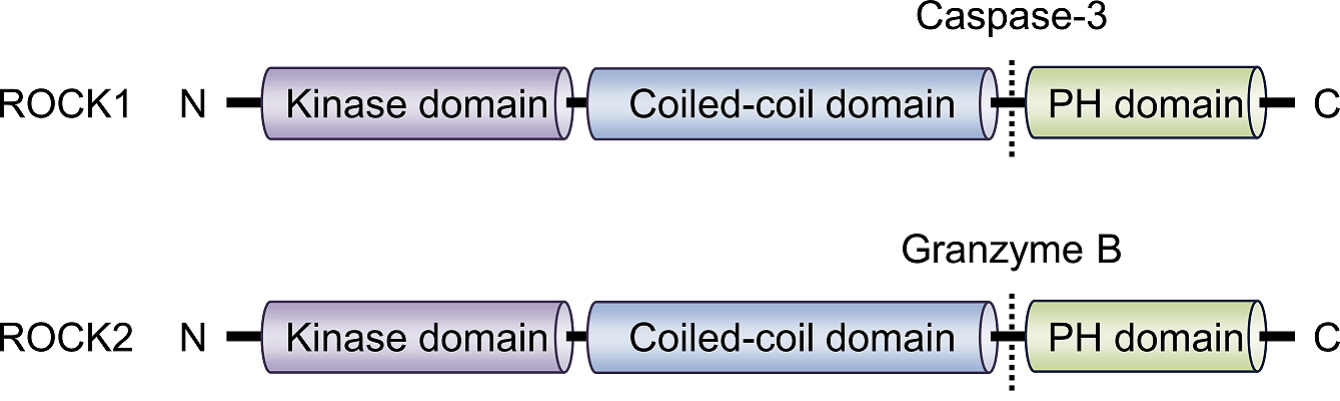
Structure of ROCK isoforms. ROCK1 and ROCK2 are known as ROKβ and ROKα, respectively. Both isoforms consist of three major domains, including a kinase domain in the N-terminal domain, a coiled-coil domain that contains a Rho-binding domain, and a putative pleckstrin homology (PH) domain at its C-terminal end.

ROCK consists of an N-terminal kinase domain, followed by a central coiled-coil-forming region containing a Rho-binding domain (RBD), and C-terminal cysteine-rich domain located within the pleckstrin homology (PH) motif. To date, two isoforms have been identified in mammals, ROCK1/ROKβ and ROCK2/ROKα (Figure 1), which are located on chromosome 18 and 2 respectively. The ROCKs share 65% overall identity (58% in the RBD and 92% in the kinase domain). Despite the high sequence homology, kinase activation is mediated through different machinery: ROCK1 is activated after the cleavage of C-terminal PH domain by caspase-3, whereas ROCK2 is activated by granzyme B-mediated cleavage.

Both isoforms are expressed ubiquitously from embryonic development to adulthood, with the predominant distribution of ROCK1 in non-neuronal tissue, such as the liver and lungs, and the predominant distribution of ROCK2 in the brain and muscle. It has long been debated whether each ROCK isoform has a distinct function. Narumiya et al. greatly advanced our understanding by showing that the systemic deletion of ROCK1 or ROCK2 results in different phenotypes. For instance, ROCK1 deficiency leads to impaired closure of the eyelids and umbilical ring in mice ^(6)^, whereas ROCK2 deficiency results in intrauterine growth retardation ^(7)^. These findings indicate that each isoform has distinct physiological and pathological roles that may vary depending on cell types.

## 3. Role of ROCK in DKD

**Figure 2.**
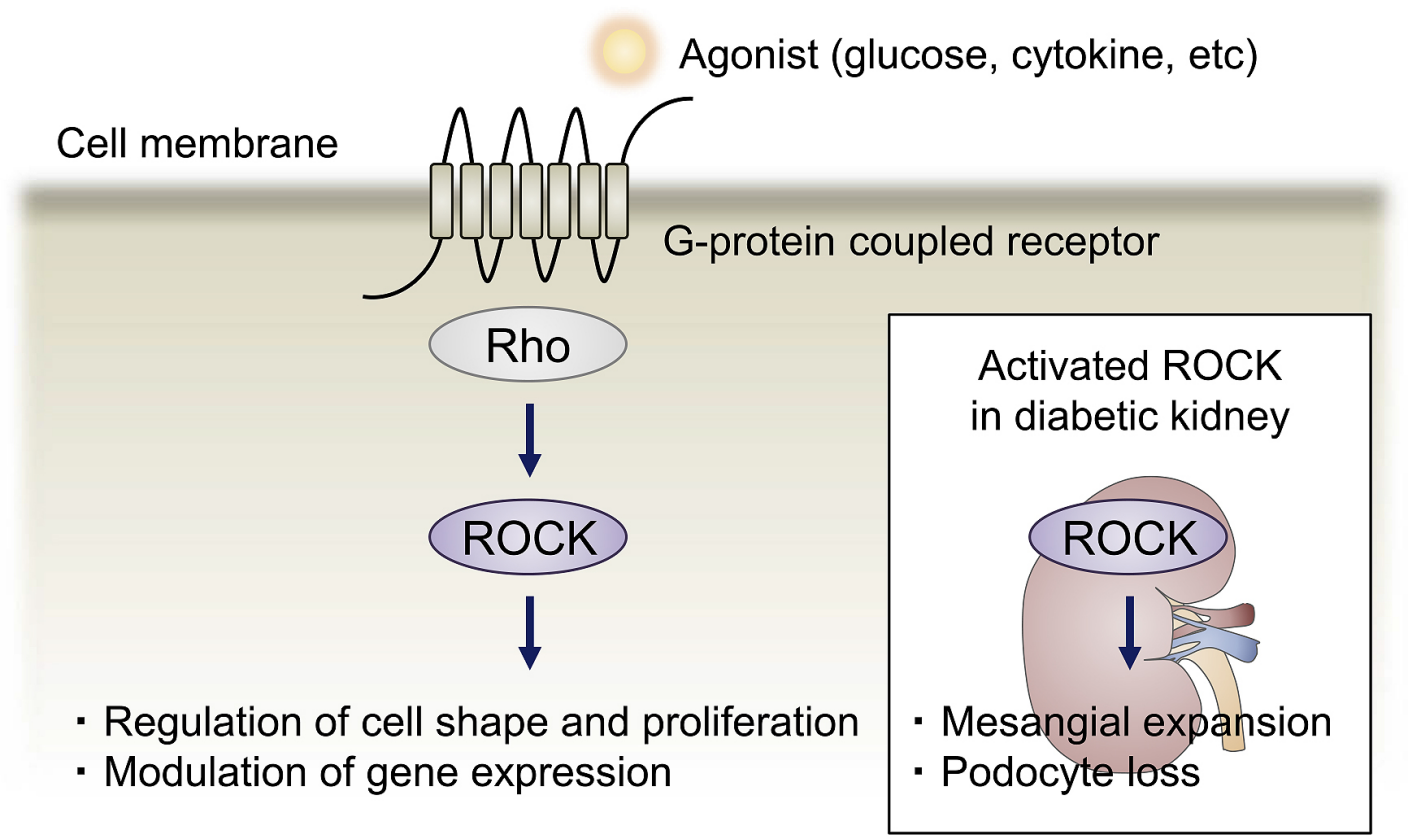
ROCK in renal health and disease. Rho/ROCK signaling executes control of the cell shape, proliferation, and gene expression via cytoskeletal rearrangement. From a pathological perspective, renal ROCK signaling is activated by glucose and cytokines in the context of diabetes. The effects of the elevated ROCK activity manifest as the progression of mesangial expansion and podocyte loss, establishing a critical role for ROCK in DKD.

Initial insights linking ROCK to diabetic vascular complications were gleaned from our studies that identified ROCK as an important molecule of the pathological changes of the microvasculature. Table 1 highlights the findings pertaining to ROCK in diabetic vascular complications. Astonishingly, elevated ROCK activities have been detected in various tissues and cells associated with diabetes, including the renal cortex, retina, neuron, and endothelium ^(8), (9), (10)^. Previous studies have linked various factors to aberrant ROCK signaling; ROCK is activated under high glucose conditions as well as by stimulation with cytokines that have implicated in insulin resistance, such as tumor necrosis factor α (TNF-α), interleukin 1β (IL-1β), and angiotensin II ^(11), (12)^. Growth factors, including platelet-derived growth factor-BB (PDGF-BB) and transforming growth factor β (TGF-β) are also known triggers ^(13), (14), (15)^. Elevated Rho/ROCK activity is implicated in the initiation of vascular inflammation, elaboration of extracellular matrix, angiogenesis, and apoptosis, all of which are involved in the pathogenesis of vascular disease in diabetes (Figure 2). By showing insights gained from *in vitro* and *in vivo* studies, we highlight the significance of ROCK in DKD and how modulation of this signaling mechanism holds therapeutic promise.

**Table 1.** Summary of Published Findings Pertaining to ROCK in Diabetic Vascular Complications.

	Function/observation	References
**Nephropathy**		
Mesangial cells	・Activated by glucose, TNF-α, TGF-β, angiotensin II, and VEGF	^(8), (11), (16), (17), (18), (65)^
	・ROCK inhibitor-treated diabetic mice exhibit reduced glomerular sclerosis and macrophage infiltration	
	・Mediates HIF1-induced fibrotic responses (CTGF, PAI-1)	
	・Regulates the expression of MCP-1 and M-CSF by controlling AP-1 and NF-κB	
Podocytes	・Activated by glucose, TGF-β, and ROS	^(14), (16), (59)^
	・ROCK inhibitor-treated diabetic rodents exhibit reduced albuminuria and apoptosis	
	・Forced expression of ROCK results in albuminuria and apoptosis	
	・Regulates the expression of Notch ligand and mitochondrial morphology	
Glomerular endothelium	・Activated by AGEs	^(66)^
	・Mediates permeability, morphology, and EMT	
	・Regulates the expression of adhesion molecules and chemokines	
Tubules	・ROCK inhibitor-treated diabetic rats exhibit reduced interstitial fibrosis	^(19), (46)^
	・Regulates sphingosine-1-phosphate-induced EMT	
**Retinopathy**		
Pericytes	・ROCK inhibitor reduces retinal VEGF expression in diabetic rats	^(49)^
	・Regulates PDGF-induced VEGF expression	
Endothelium	・ROCK inhibitor reduces leukocyte adhesion and the number of damaged cells in diabetic rats
	・Regulates the expression of ICAM1	
**Neuropathy**		
Myelin sheath	・ROCK inhibitor treated diabetic rats exhibit improvement of motor nerve conduction velocity	^(10)^
	・Regulates distribution of adhesion molecules	
	・Pathogenic roles in other cell types in nervous system in not determined	
**Large vessels**		
VSMC	・Activated by static pressure, angiotensin II, thrombin, PDGF, extracellular nucleotides, and urotensin	^(13), (54)^
	・Mediates cell proliferation, migration, Ca^2+^ sensitization, and contraction	
	・Regulates the expression of IL-6, MCP-1, MIF, ROS formation, and cyclophilin A secretion	
Endothelium	・Activated by angiotensin II, IL-1β, thrombin, ER stress, and lysophosphatidic acid	^(38), (54), (56)^
	・Mediates barrier function and permeability	
	・Regulates the expression of E-selectin, MCP-1, VCAM1, NO production, NADPH oxidase activity	
Inflammatory cells	・Regulates chemotaxis and foam cells formation by inhibiting reverse cholesterol transport	^(63), (67)^

TNF-α, tumor necrosis factor α; TGF-β, transforming growth factor β; VEGF, vascular endothelial growth factor; MCP-1, Monocyte chemoattractant protein 1; M-CSF, macrophage colony-stimulating factor; AP-1, activator protein 1; NF-κB, nuclear factor κB; HIF-1, hypoxia-inducible factor 1; CTGF, connective tissue growth factor; PAI-1, plasminogen activator inhibitor 1; ROS, Reactive oxygen species; AGEs, advanced glycation end products; EMT, epithelial mesenchymal transition; PDGF, platelet-derived growth factor; ICAM1, intercellular adhesion molecule 1; IL-6, interleukin 6; MIF, macrophage migration inhibitory factor; ER, endoplasmic reticulum; IL-1β, interleukin 1β; VCAM1, vascular cellular adhesion molecule 1; NO, nitric oxide; NADPH, nicotinamide adenine dinucleotide phosphate.

### 3.1. ROCK-mediated diabetic glomerular sclerosis

**Figure 3.**
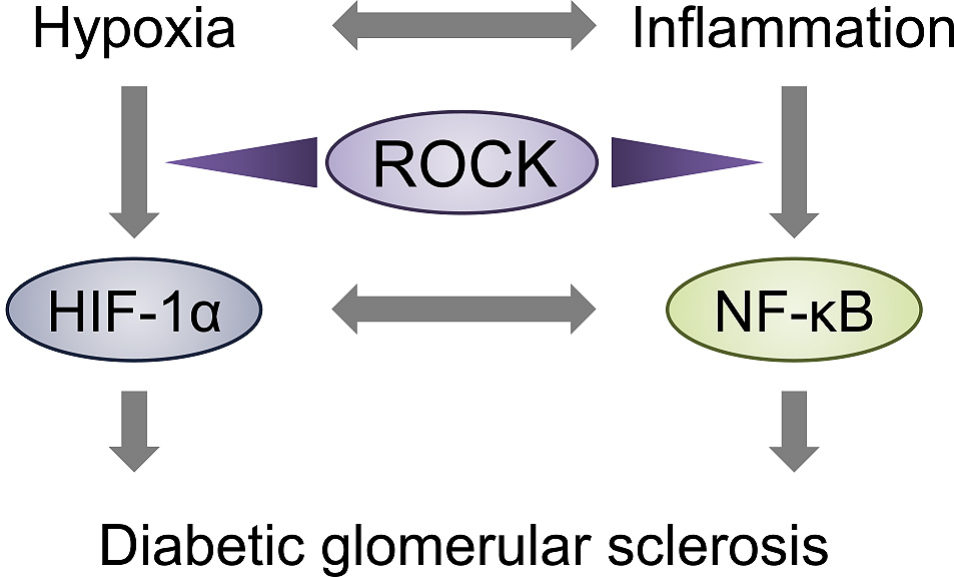
ROCK-dependent transcriptional regulation during the progression of DKD. A parallel mechanism operates in the diabetic kidney via hypoxia-dependent fibrotic responses and tissue damage elicited by inflammation. ROCK governs two important transcription factors in this framework, hypoxia-inducible factor 1α (HIF-1α) and nuclear factor κB (NF-κB). The molecular basis for these effects involves the inhibition of proteasomal degradation of HIF-1α and promotion of NF-κB nuclear transport.

In renal tissue, a broad range of cell types are damaged under the diabetic milieu. Traditionally, the most remarkable morphological abnormality in DKD is nodular glomerulosclerosis (also known as Kimmelstiel-Wilson syndrome) caused by glomerular mesangial expansion. Under homeostatic conditions, the fundamental function of the mesangial cells is the regulation of glomerular filtration and structural support of capillaries. In response to injury, mesangial cells assume a different phenotype characterized by the overproduction of extracellular matrix components such as fibronectin, type IV collagen, and laminin. While this response is likely adaptive, an exaggerated action can contribute to the development of glomerular sclerosis. We and others established the pathological relevance by showing that the pharmacological inhibition of ROCK, when administered orally to the streptozotocin-induced type 1 diabetic rat, not only prevented albuminuria but also attenuated mesangial expansion and the overproduction of fibrotic mediators ^(16), (17)^. ROCK blockade is also effective for the prevention of progressive glomerulosclerosis in type 2 diabetic db/db mice ^(8), (18)^. These beneficial renal outcomes were independent of glycemic control; however, ROCK inhibitor treatment improves metabolic parameters when administered at a high dose (100 mg/kg) ^(19)^. One important mechanism underlying the renoprotective actions of ROCK inhibition is the regulation of hypoxia-induced inflammation and vice versa (Figure 3).

Renal hypoxia has been implicated in the initiation and worsening of several kidney diseases including DKD ^(20), (21), (22)^, ^(23)^. Hypoxia-inducible factor 1 (HIF-1), a basic helix-loop-helix transcription factor that consists of an oxygen-sensitive α-subunit and a constitutively expressed β-subunit, is a key mediator of oxygen homeostasis. In both animal models and patients with DKD, HIF-1α has been detected in the tubulointerstitium, particularly in the medullary region, where oxygen tension is known to be low ^(24)^. In addition, the expression of HIF-1α is evident in glomerular mesangial cells in the setting of diabetes ^(25)^. Glucose is known to elicit the expression of HIF-1α in the mesangium, which eventually converges to transactivate downstream fibrotic factors, such as connective tissue growth factor (CTGF) and plasminogen activator inhibitor 1 (PAI-1), which are known to be involved in glomerular fibrosis. In experimental models of DKD, ROCK inhibition blunts the development of mesangial expansion, albuminuria, and glomerular hypertrophy without affecting metabolic factors (*i.e.*, body weight, blood glucose levels, and blood pressure) via the downregulation of HIF-1α ^(8)^. Based on the finding that ROCK inhibitor-mediated HIF-1α degradation is canceled by proteasome inhibitor, and that the polyubiquitination of HIF-1α is promoted by ROCK inhibition, it is conceivable that the proteolysis is mediated by the ubiquitin-proteasome pathway. Given the critical role of HIF-1α in tubulointerstitial fibrosis and chronic kidney disease (CKD) ^(26)^, a robust appreciation of the role of tubular ROCK will be required in future.

Recent studies have implicated hypoxia-elicited inflammation or inflammation in the context of hypoxic states in various human diseases. Macrophage infiltration is detected in CKD, including DKD, and is correlated with pathological changes and the prognosis of the renal function. The interaction of monocytes and cell adhesion molecules, mediated by chemokines and cytokines, is crucial for the recruitment of circulating monocytes into the sites of renal inflammation.

Monocyte chemoattractant protein 1 (MCP-1) plays a key role in the recruitment of macrophages to renal tissue. Chow et al. investigated the progression of DKD in streptozotocin-injected MCP-1-null mice in order to characterize the role of MCP-1-mediated macrophage accumulation in the development of DKD ^(27)^. In MCP-1 deficient mice, albuminuria was decreased in comparison to wild-type mice, and the protection effect was associated with marked reductions in glomerular and interstitial macrophage accumulation. Moreover, macrophage colony-stimulating factor (M-CSF) is required for macrophage proliferation throughout the G1 phase of the cell cycle ^(28)^ and is detected in glomerular mesangial cells, tubular epithelial cells, and endothelial cells ^(29), (30), (31)^. Consistently, the genetic deletion of M-CSF in mice leads to the attenuation of macrophage infiltration and local proliferation in the process of renal inflammation ^(32)^. Furthermore, the implantation of M-CSF-producing cells into the kidneys of autoimmune lupus mice induced local inflammation ^(33)^. In stark contrast, treatment with antibodies against the M-CSF receptor inhibits inflammation in animal models of kidney disease, such as unilateral ureteric obstruction and DKD ^(34), (35)^. Collectively, these findings suggest an essential role of MCP-1 and M-CSF in inflammatory reaction in renal diseases.

To glean insights into the potential of ROCK operating inflammation in DKD, we assessed the contribution of ROCK on the production of these chemokines. Of note, pro-inflammatory TNF-α, a cytokine secreted from adipose tissue in the obese state, induces the mesangial expression of MCP-1 and M-CSF in a ROCK-dependent manner ^(11), (12)^. Consistent with these data, macrophage accumulation is attenuated in the glomeruli of ROCK inhibitor-treated db/db mice. Mechanistically, ROCK and its downstream target, p38 MAPK, mediate the nuclear uptake of nuclear factor κB (NF-κB), a well-characterized transcription factor activator of inflammatory reactions. Increased NF-κB levels are detected in the kidneys of diabetic experimental models, which activate glomerular and tubular cells to induce renal inflammation ^(11), (36)^. The primary downstream mechanism by which NF-κB carries out its pro-inflammatory effects is via the enhancement of DNA binding. The targets of NF-κB include adhesion molecules and cytokines (*e.g.*, interleukin 6, TNF-α), which all drive oxidative stress and the development of DKD. The mechanistic link between ROCK and NF-κB has been identified in other models of renal injury and in other cell types ^(37)^. For example, thrombin and lysophosphatidic acid are known to activate endothelial NF-κB in a ROCK-dependent manner ^(38), (39)^. Under physiological settings, NF-κB is sequestered in the cytoplasm by IκBα. By the phosphorylation, IκBα is ubiquitinated and degraded in a proteasome-dependent fashion. The subsequent recognition of NF-κB by karyopherin β directs it to the nuclear pore complex, where the nuclear uptake takes place. While some studies reported a regulatory role of ROCK in IκBα degradation, mesangial ROCK regulates NF-κB without affecting this step. Therefore, it is reasonable to conclude that ROCK is a critical determinant of renal inflammation *in vivo* and *in vitro*, and future studies aimed at elucidating the interaction between ROCK and other transcriptional circuitry or inflammasome activation will prove beneficial.

### 3.2. ROCK inhibition for podocytopathy in DKD

Since glomerular podocytes are highly differentiated cells and have a limited ability to self-repair and regenerate, podocyte damage is an essential factor in determining the prognosis of DKD. Podocyte foot processes are interconnected by slit diaphragms and form the glomerular final filtration barrier. Nephrin and podocin are important plasma-membrane proteins in the formation of the slit diaphragm. Pathological changes of podocyte foot processes, such as effacement and apoptosis, are hallmarks of DKD, and Notch signaling has been implicated as a key determinant of this process ^(40), (41)^. Physiologically, Notch signaling regulates the cell fate during kidney development, including nephron segmentation and endowment. Notch receptors are a family of transmembrane proteins that require cell-cell interaction in order to be activated, and two families of Notch ligands, namely Jagged-like and Delta-like, are reported in all metazoans. Upon the binding of Notch receptors to Notch ligands, C-terminal Notch intracellular domain is released from the cell membrane by γ-secretase and travels to the nucleus, which then forms a complex with the recombination signal binding protein for immunoglobulin κJ region (Rbpj) and mastermind-like proteins to initiate target gene transcription ^(42)^.

In the context of diabetes, renal Notch signaling is reactivated in both type 1 and type 2 diabetic mice to induce the expression of Notch ligands and Rbpj-dependent transcriptional activation of Notch targets ^(43)^. Exposure to high glucose, TGF-β, or vascular endothelial growth factor (VEGF) could be inducers of the Notch pathway ^(44)^. The inhibition of pathogenic Notch signaling has been shown to alter the natural history of DKD. Interestingly, ROCK mediates the TGF-β-induced Notch ligand expression and podocyte survival ^(14)^. From a mechanistic standpoint, ROCK regulates the induction of Jagged-like ligand via the extracellular signal-regulated kinase (ERK) 1/2 and c-Jun N-terminal kinase (JNK) but not Smad signals. Consistent with these findings, ROCK inhibitor attenuates the urinary excretion of albumin and podocyte apoptosis in db/db mice. Benefits of ROCK inhibition have also been reported in type 1 diabetic rats ^(16)^. Cumulatively, these data support the hypothesis that ROCK-Notch molecular axis coordinates the expression of genes essential for podocyte survival and that the inhibition of overactive ROCK in podocytes could be a promising therapeutic opportunity.

### 3.3. Diabetic tubular injury and ROCK

In addition to glomerular injury, tubulointerstitial damage is becoming increasingly recognized as a significant component of DKD pathology. An elegant study from the Shimokawa Laboratory demonstrated the contribution of ROCK to interstitial fibrosis using rats with unilateral ureteral obstruction ^(45)^. They reported that macrophage infiltration and interstitial sclerosis were significantly inhibited in animals treated with a ROCK inhibitor. We have previously shown that ROCK mediates the epithelial mesenchymal transition (EMT) induced by sphingosine-1-phophate, a bioactive sphingolipid ^(46)^. Furthermore, the combination of an angiotensin-converting enzyme inhibitor and ROCK inhibitor is more effective in comparison to either agent alone for the treatment of interstitial fibrosis through the attenuation of inflammation and the oxidative stress pathway ^(47)^. The ROCK signal has also been implicated in other renal diseases, such as hypertensive glomerulosclerosis and subtotal nephrectomy. Given the significance of tubulointerstitial fibrosis as the common final pathway in CKD, ROCK breakdown may be effective in the treatment of a broad range of kidney diseases and the prevention of renal failure.

## 4. ROCK in Other Diabetic Vascular Complications

### 4.1. The involvement of ROCK in diabetic retinopathy

Vitreoretinal diseases, including diabetic retinopathy and age-related macular degeneration, are still a major cause of blindness. In the area of ophthalmology, the beneficial effects of ROCK inhibition were originally discovered by Honjo et al. ^(48)^. They demonstrated that ROCK inhibition reduced the intraocular pressure. Based on these pre-clinical findings, coupled with the work of others, a selective ROCK inhibitor, ripasudil hydrochloride hydrate (K-115), was clinically approved in 2014 as an eye drop for glaucoma in Japan. Moreover, the intravitreal administration of a ROCK inhibitor significantly reduced leukocyte adhesion as well as the number of damaged endothelial cells in the retinas of diabetic rats ^(9)^. Yokota et al. demonstrated that, in comparison to untreated streptozotocin-induced diabetic rats, ROCK inhibitor significantly attenuated the retinal expression of VEGF, the major factor responsible for neovascularization in diabetic retinopathy ^(49)^. Moreover, in both rodents and patients with diabetes, ROCK is activated and associated with membrane blebbing in retinal vessels and pigment epithelium ^(50)^. Notably, the intravitreous injection of a ROCK inhibitor restored the retinal pigment epithelium structure and barrier function. ROCK inhibition is also effective for the prevention of fibrosis and neovascularization seen in age-related macular degeneration ^(9)^. These data suggest the therapeutic potential of ROCK inhibitor treatment in eye disease.

### 4.2. ROCK as a possible target in diabetic neuropathy

Currently available therapies for diabetic neuropathy are aimed at improving glycemic control and the management of paresthesia. There is no causal therapy for diabetic neuropathy. Experimental work has revealed the mechanistic basis of the interplay between ROCK and diabetic neuropathy. Kanazawa et al. reported that the neural function, examined based on the motor nerve conduction velocity, was maintained in diabetic rats treated with ROCK inhibitor ^(10)^. It is important to note that ROCK inhibition prevented slowing of motor nerve conduction velocity by altering the expression pattern of adhesion molecules in the myelin sheath in diabetic rats. While previous findings implicate a ROCK-dependent mechanism in the context of brain stem dysfunction ^(51)^, brain axonal regeneration ^(52)^, and sexual dysfunction ^(53)^, this report is the first to demonstrate that ROCK is required for diabetic peripheral nerve injury. Future experiments should focus on the role of ROCK in mediating diabetes-related gene programs in nerves.

### 4.3. Role of ROCK in macrovascular complications

Diabetes accelerates atherosclerosis, which is an underlying condition in the majority of patients with occlusive vascular diseases; thus, it is important to understand the molecular machinery. Vascular ROCK has been a subject of intensive exploration in human health and disease, and there is now satisfactory evidence to illustrate the causal link between ROCK and the progression of atherosclerosis. Recent studies have shown that ROCK activation in vascular smooth muscle cells (VSMCs) and endothelial cells is the essential step in the process of cerebrovascular and ischemic heart diseases, such as ischemic stroke or vasospastic angina^(13)^, ^(38)^, ^(54)^. Clinically, ROCK is believed to contribute to some of the lipid lowering-independent cardiovascular benefits seen in patients treated with statins (*i.e.*, pleiotropic effects) ^(17), (55)^.

At the cellular level, ROCK regulates many molecules associated with proliferation, inflammation, thrombosis, and fibrosis. Our group identified ROCK as an essential regulator of the proliferation of VSMCs induced by PDGF-BB ^(38)^. With respect to endothelial cells, Kawanami et al. showed that ROCK inhibition attenuates the vascular cellular adhesion molecule 1 (VCAM1) expression induced by tunicamycin, an inducer of endoplasmic reticulum (ER) stress ^(56)^. Mechanistically, activating transcription factor 4 (ATF4) and C/EBP homologous protein (CHOP), which are involved in ER stress-induced cellular apoptosis, are downregulated by the ROCK inhibition. As was the case in renal mesangial cells ^(12)^, these actions are mediated by p38 MAPK, suggesting that p38 MAPK is requisite for the ability of ROCK to induce a subset of target genes that are critical for tissue damage.

For the past two decades, translational research studies have established the essential roles of ROCK in the pathogenesis of cardiovascular diseases ^(57)^. In Japan and China, intravenous infusion of fasudil, a potent inhibitor of ROCK, has been approved for short-term clinical use for the prevention of cerebral vasospasm and subsequent ischemic injury in patients with subarachnoid hemorrhage. The development of oral fasudil will continue to pave the way for novel cardiovascular therapeutics.

## 5. ROCK Isoform Selectivity in DKD

The specific actions of the ROCK isoform in the kidney and other tissues to induce diabetic vascular complications are just beginning to be undertaken using pharmacological and gene targeting approaches in rodents. In mice, both ROCK1 and ROCK2 are detected in the kidney ^(58)^; however, the detailed cellular distribution and function of each isoform are not well understood.

In an effort to parse the role of ROCK isoform in renal biology *in vivo*, the ROCK1 gene was targeted through cell-specific approaches. The importance of podocyte ROCK1 in DKD was first described by Wang et al. ^(59)^. The exposure of podocytes to high glucose results in a significant elevation of ROCK1 activity, indicating a potential role for ROCK1 in the podocytopathy seen in diabetes. Mechanistically, ROCK1 regulates mitochondrial fission by phosphorylating Drp1 at serine residue 600. In addition, glomerular endothelial ROCK1 has been demonstrated to regulate EMT, which can cause loss of tight junctions and albuminuria in DKD ^(60)^. These exciting data indicate that ROCK1 is involved in hyperglycemia-induced aberrant functions in slit diaphragm at early stages of DKD. Further mechanistic investigations are required in order to validate the role of ROCK1 in the progressive stages of DKD.

While no specific inhibitor of ROCK1 has been developed, ROCK2 has been extensively studied with a ROCK2 inhibitor, KD025 (formerly known as SLx-2119). Radiometric enzyme assays confirmed that KD025 selectively inhibits ROCK2 activity (IC50 = 105 nM), whereas the effects on ROCK1 were minimal (IC50 = 24 μM) ^(61)^. Nagai et al. found that the ROCK2 activity is elevated in the renal cortex of db/db mice, and inhibition of its activity with SLx-2119 significantly attenuated the increased albuminuria and improved histological abnormalities in the diabetic mice ^(15)^. Moreover, technologies, such as transfection of renal cells with small interfering RNA targeting ROCK2 isoform, have provided valuable information on ROCK2 isoform specificity. For example, ROCK2-null endothelium is characterized by a significant reduction in adhesion molecules and inflammatory cytokines, with a concomitant decrease in monocyte recruitment ^(62)^. An appreciation of the pathological importance of cardiovascular ROCK2 principally developed through studies in the Liao Laboratory. The study suggests that, in large vessels, ROCK2 contributes cell formation by attenuating peroxisome proliferator-activated receptor-γ-mediated reverse cholesterol transport in macrophages ^(63)^. Moreover, ROCK2 regulates cardiomyocyte hypertrophy and apoptosis through interaction with serum response factor and ERK ^(64)^. These observations coupled with the work of others, have led to an increasing appreciation that ROCK2 is a nodal determinant of renal and cardiovascular complications.

## 6. Conclusions and Future Perspectives

The mortality of DKD is still high, and the associated healthcare costs are increasing worldwide. From this perspective, renal protection is a critical issue in the management of diabetes. Therapeutic strategies against DKD are currently limited to intensive intervention for metabolic risk factors, mainly with the use of RAS inhibitors. We and others have provided the first evidence implicating ROCK as an essential regulator in the progression of DKD as well as in diabetic retinopathy, neuropathy, and cardiovascular disease. With the development of specific ROCK inhibitors (*e.g.*, fasudil and Y-27632), research on ROCK has garnered significant interest and is greatly expanding. When it is considered alongside the fact that ROCK signaling is important in various pathological aspects of renal injury and other micro and macrovascular complications, the blockade of ROCK signaling could have therapeutic value for the improvement of quality of life in patients with diabetes. Long-term studies are required with new oral compounds to assess the safety profile of ROCK inhibitors. With regard to the isoform specificity, strategies to target ROCK1 or ROCK2 may be a promising option. However, whether it is better to inhibit each ROCK isoform or both is still debated. Conditional gene targeting and inducible transgenes will be required to dissect the divergent and redundant roles of ROCK in renal biology *in vivo*. Most importantly, future studies are necessary to determine whether ROCK isoform inhibition is a clinically translatable approach in the setting of DKD.

## Article Information

### 

This article is based on the study, which received the Medical Research Encouragement Prize of The Japan Medical Association in 2019.

### Conflicts of Interest

Keiichiro Matoba received research support from Sanofi KK, Tanabe Pharma, and Takeda Pharmaceutical; Daiji Kawanami received research support from Sanofi KK, Tanabe Pharma, Terumo, Böehringer Ingelheim, Kyowa Hakko Kirin, Sumitomo Dainippon Pharma, Ono Pharmaceutical, and Takeda Pharmaceutical, as well as speaker honoraria from Novo Nordisk Pharma, Sanofi KK, and Takeda Pharmaceutical; Rimei Nishimura received speaker honoraria from Astellas Pharma, Nippon Boehringer Ingelheim, Eli Lilly Japan KK, Kissei Pharmaceutical, Medtronic Japan, MSD, Novartis Pharma KK, Novo Nordisk Pharma, Sanofi KK, and Takeda Pharmaceutical.

### Sources of Funding

This work was supported by a Grant-in-Aid for Scientific Research from Japan Society for the Promotion of Science (to Keiichiro Matoba, Daiji Kawanami, and Rimei Nishimura), the MSD Life Science Foundation (to Keiichiro Matoba), the Takeda Science Foundation (to Keiichiro Matoba), the Suzuken Memorial Foundation (to Keiichiro Matoba), and the Japan Diabetes Foundation (to Rimei Nishimura).

### Acknowledgement

Some of the contents of this review were generated based upon works of authors (reference number 8, 10, 11, 14, 15, 47).

### Author Contributions

Keiichiro Matoba wrote the manuscript. Yusuke Takeda, Yosuke Nagai, Yasushi Kanazawa, Daiji Kawanami, Tamotsu Yokota, Kazunori Utsunomiya, and Rimei Nishimura helped edit and revised the manuscript for important intellectual content. All of the authors approved the final manuscript.
